# How Priming Exercise Affects Oxygen Uptake Kinetics: From Underpinning Mechanisms to Endurance Performance

**DOI:** 10.1007/s40279-023-01832-1

**Published:** 2023-04-03

**Authors:** Richie P. Goulding, Mark Burnley, Rob C. I. Wüst

**Affiliations:** 1 Laboratory for Myology, Faculty of Behavioural and Movement Sciences, Amsterdam Movement Sciences, Vrije Universiteit Amsterdam, De Boelelaan 1108, 1081 HZ Amsterdam, The Netherlands; 2 School of Sport, Exercise and Health Sciences, Loughborough University, Loughborough, UK

## Abstract

The observation that prior heavy or severe-intensity exercise speeds overall oxygen uptake ($\dot{V}$O_2_) kinetics, termed the “priming effect”, has garnered significant research attention and its underpinning mechanisms have been hotly debated. In the first part of this review, the evidence for and against (1) lactic acidosis, (2) increased muscle temperature, (3) O_2_ delivery, (4) altered motor unit recruitment patterns and (5) enhanced intracellular O_2_ utilisation in underpinning the priming effect is discussed. Lactic acidosis and increased muscle temperature are most likely not key determinants of the priming effect. Whilst priming increases muscle O_2_ delivery, many studies have demonstrated that an increased muscle O_2_ delivery is not a prerequisite for the priming effect. Motor unit recruitment patterns are altered by prior exercise, and these alterations are consistent with some of the observed changes in $\dot{V}$O_2_ kinetics in humans. Enhancements in intracellular O_2_ utilisation likely play a central role in mediating the priming effect, probably related to elevated mitochondrial calcium levels and parallel activation of mitochondrial enzymes at the onset of the second bout. In the latter portion of the review, the implications of priming on the parameters of the power–duration relationship are discussed. The effect of priming on subsequent endurance performance depends critically upon which phases of the $\dot{V}$O_2_ response are altered. A reduced $\dot{V}$O_2_ slow component or increased fundamental phase amplitude tend to increase the work performable above critical power (i.e. W´), whereas a reduction in the fundamental phase time constant following priming results in an increased critical power.

## Key Points

**Table:** 

A prior bout of heavy-intensity exercise speeds pulmonary oxygen uptake kinetics during subsequent exercise; however, the physiological mechanisms underpinning this effect have been the subject of great debate.
We critically evaluate the literature from the past 25 years, and conclude that this effect is primarily mediated by enhanced intracellular oxygen utilisation and altered motor unit recruitment patterns.
We further provide recommendations for the role of priming exercise in enhancing endurance performance.

## Introduction

In 1996, it was demonstrated that pulmonary oxygen uptake ($\dot{V}$O_2_) kinetics were speeded when heavy-intensity exercise was preceded by a prior bout of a heavy-intensity warm-up [1]. This phenomenon was termed the “priming” effect [1]. Since this seminal paper, the physiological mechanisms underpinning the effect of priming on subsequent $\dot{V}$O_2_ kinetics has received considerable research attention, not least because of the ability of priming to enhance subsequent endurance exercise performance [2–6]. The physiological mechanisms underpinning the effect of prior exercise on $\dot{V}$O_2_ kinetics and subsequent performance, however, are difficult to pinpoint as priming upregulates multiple steps in the O_2_ transport and utilisation pathways. As a result, the primary factors responsible are still a matter of intense debate [7–9]. The purpose of this review is to summarise the advancements in understanding that have been made since the 2003 review in this journal on the same topic [10]. In particular, this review focuses on disentangling the factors that contribute to this effect and how endurance performance is enhanced by prior exercise.

### $\dot{V}$O_2_ Kinetics Response to Exercise

During the transition from a lower to a higher metabolic rate, the increase in muscle O_2_ consumption lags behind the increase in muscle ATP utilisation with near-exponential kinetics. Any energy provision not met via oxidative phosphorylation during the transition phase is met via non-oxidative sources (i.e. phosphocreatine [PCr] breakdown and glycolysis). The degree of reliance on non-oxidative sources of energy provision during the rest-to-work transition— termed the O_2_ deficit [11]—is associated with the build-up of fatigue-related metabolites [12–15] and the breakdown of finite muscle fuel reserves such as glycogen and PCr [16, 17]. Hence, a faster increase in muscle $\dot{V}$O_2_ at exercise onset predisposes towards better exercise tolerance because reliance on substrate-level phosphorylation is minimised [2, 18, 19], and as a result, the degree of fatigue incurred is reduced [12–14, 20].

**Fig. 1:**
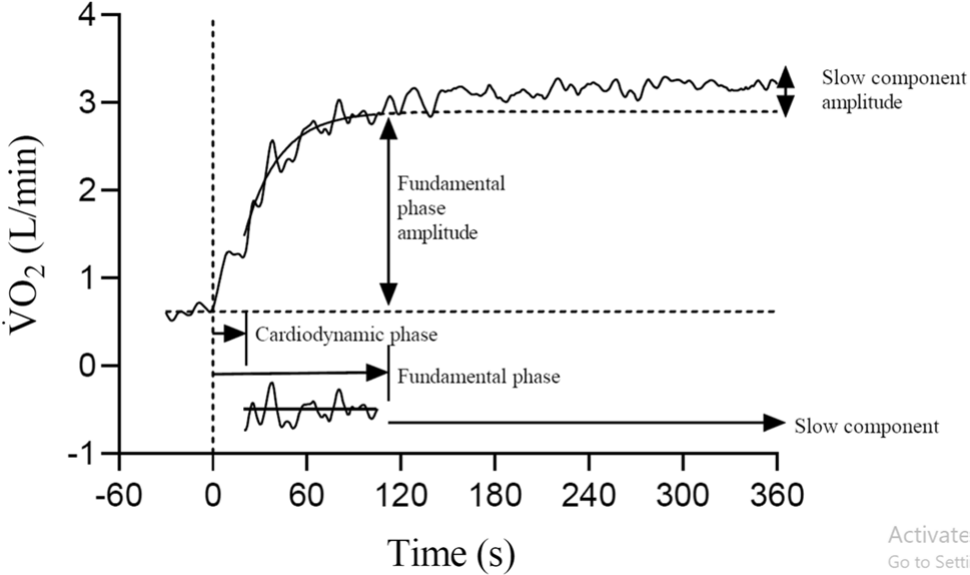
Typical pulmonary oxygen uptake ($\dot{V}$O_2_) response to a 6-min bout of heavy-intensity exercise. The cardiodynamic phase is followed by the fundamental phase, which is in turn followed by the slow component. The slow component emerges from the fundamental phase some 90–180 s after exercise onset (106 s in this case). Residuals of the fundamental phase fit are displayed at the bottom

Figure 1 provides a schematic overview of the phases that occur during the transition from rest to exercise. When measured at the mouth, the exponential rise in pulmonary $\dot{V}$O_2_ closely reflects that of muscle $\dot{V}$O_2_ (i.e. the fundamental phase [21–23]) once the deoxygenated blood draining the muscle reaches the lungs, i.e. following a time delay termed the cardiodynamic phase [24, 25]. The fundamental phase is characterised by a time constant ($\tau_{{\dot{V}{\text{O}}_{2} }}$), which reflects the time taken to attain 63% of the final steady-state value, with a gain that approximates 10 mL.min^−1^.W^−1^ at moderate intensities (i.e. below the lactate threshold). Values for $\tau_{{\dot{V}{\text{O}}_{2} }}$ vary widely between individuals, being lesser (i.e. faster $\dot{V}$O_2_ kinetics) in trained individuals and greater (i.e. slower $\dot{V}$O_2_ kinetics) in the untrained [26–30]. Above the lactate threshold, $\dot{V}$O_2_ kinetics are supplemented by a delayed increase in $\dot{V}$O_2_ that elevates the gain above that observed during moderate exercise (i.e. increasing the gain up to 14–15 mL.min^−1^.W^−1^) [31–34]. This is termed the “slow component”, a phase that emerges 90–180 s after the onset of exercise [35] and coincides with reductions in muscle PCr [16, 36] and glycogen levels [37]. During heavy-intensity exercise (above the lactate threshold but below critical power), $\dot{V}$O_2_ eventually reaches a delayed and elevated steady state [32], whereas during severe exercise (above the maximal metabolic steady state, often inferred via critical power), the slow component drives $\dot{V}$O_2_ towards its maximally attainable value ($\dot{V}$O_2_ max), with task failure occurring shortly thereafter. The mechanisms underpinning the $\dot{V}$O_2_ slow component remain somewhat unclear [20, 34, 38, 39], and are the subject of comprehensive review elsewhere [34].

## The “Priming Effect”

**Fig. 2:**
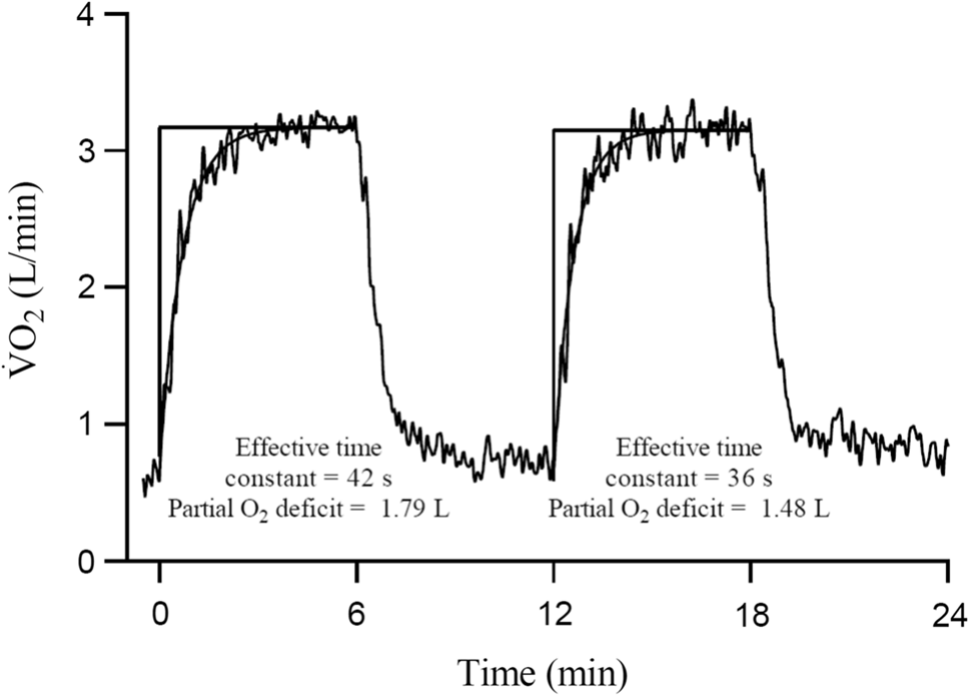
Representative oxygen uptake ($\dot{V}$O_2_) response to two identical 6-min bouts of heavy exercise, separated by 6 min of cycling at 20 W. Analysis was performed according to the original protocol of Gerbino et al. [1], that is, by fitting a single monoexponential function to the entire $\dot{V}$O_2_ response in each bout. Note the considerable speeding of the overall monoexponential kinetics in bout 2 compared with bout 1, alongside a reduction in the partial oxygen deficit. However, whether this reduction was due to a “true” speeding of the fundamental phase kinetics or to an alteration in the fundamental or slow-phase amplitudes could not be discerned from this study

**Fig. 3:**
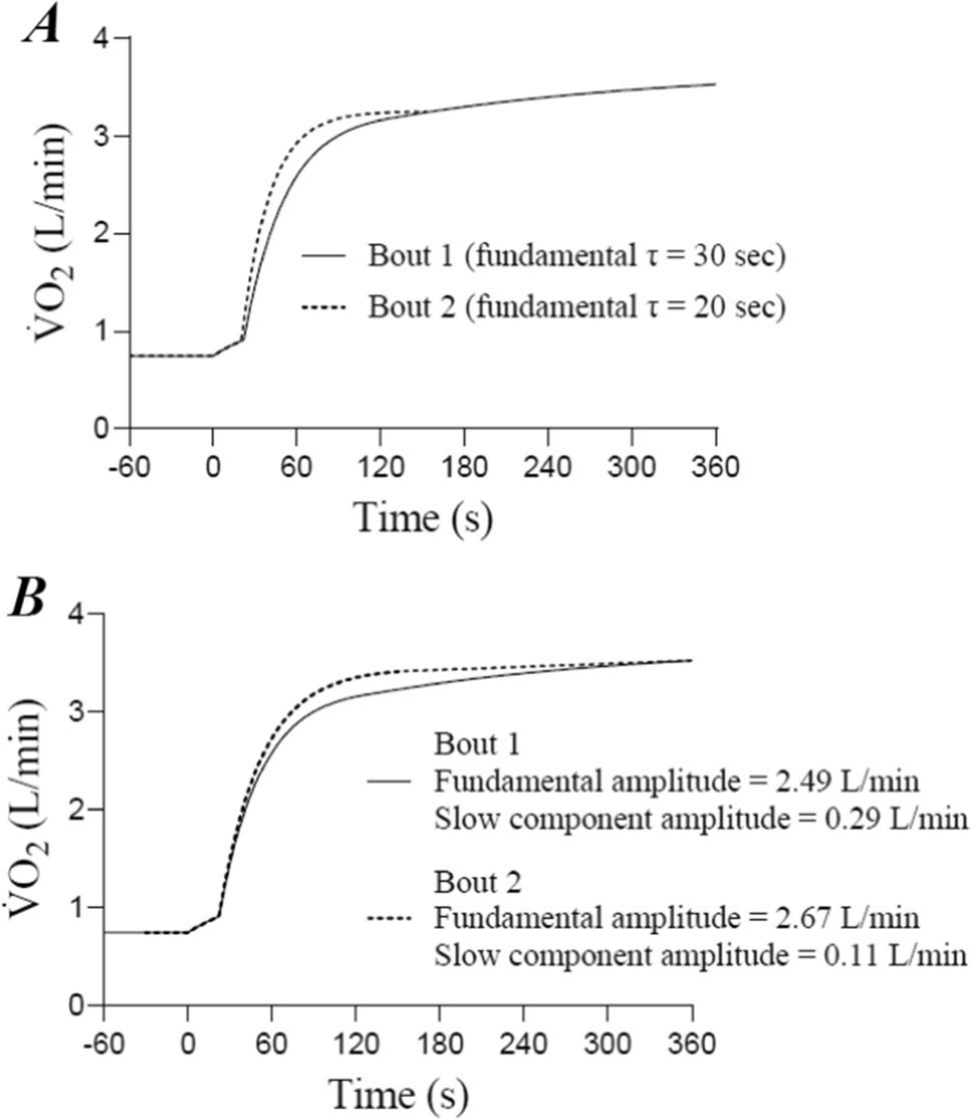
Schematic displaying the two different types of priming effect that may occur during two consecutive bouts of heavy exercise. Specifically, prior heavy exercise may cause either a speeding of the fundamental phase kinetics (**A**), or an increase in the fundamental phase amplitude and concomitant reduction in the slow component amplitude, with no effect on the speed of the kinetics (**B**). In panel **A**, the fundamental phase amplitude is identical (2.49 L/min), but the kinetics are faster enabling a more rapid attainment of the steady-state value in bout 2. In panel **B**, the fundamental phase time constant is the same (*τ*, 30 s), whereas the amplitude of the fundamental phase is increased and the slow component amplitude is reduced in bout 2. Because the fundamental phase time constant is unaltered in bout 2 in panel (**B**), this response does not represent a true speeding of the fundamental $\dot{V}$O_2_ kinetics. Both types of response have been observed in the literature following prior heavy exercise, with individuals with slower initial fundamental phase $\dot{V}$O_2_ kinetics being more likely to display the responses shown in panel (**A**) than individuals with rapid $\dot{V}$O_2_ kinetics

Early work generally showed that prior heavy exercise increased pulmonary $\dot{V}$O_2_ at a given time point during subsequent exercise [40, 41]. Gerbino et al. [1] were the first to demonstrate that a prior 6-min bout of heavy exercise speeded overall $\dot{V}$O_2_ kinetics during a subsequent bout of identical heavy exercise performed 6 min after the first (i.e. effective time constant reduced from 65 s in bout 1 to 56 s in bout 2). Importantly, the heavy exercise $\dot{V}$O_2_ kinetics were unaltered by prior moderate exercise, and prior heavy exercise had no effect on subsequent moderate exercise [1]. The authors concluded that pulmonary $\dot{V}$O_2_ kinetics were limited by O_2_ delivery during the initial bout of heavy, but not moderate exercise, and that prior heavy exercise alleviated this O_2_ delivery limitation, thereby speeding the $\dot{V}$O_2_ kinetics [1]. MacDonald et al. [42] subsequently demonstrated that both prior heavy exercise and hyperoxia both speeded the overall $\dot{V}$O_2_ kinetics during subsequent heavy (but not moderate) intensity exercise (i.e. mean response time reduced from 53 to 46 s in normoxia and 41–38 s in hyperoxia in bouts 1 and 2, respectively). Moreover, $\dot{V}$O_2_ kinetics during subsequent heavy exercise was fastest when hyperoxia was combined with prior heavy exercise, indicating that when more O_2_ was supplied to the muscle, the muscle was able to utilise it [42]. In both studies, the authors utilised a single exponential function to characterise the $\dot{V}$ O_2_ responses to heavy exercise, which included the occurrence of the slow component [1, 39]. Figure 2 illustrates this fitting strategy in a typical pulmonary $\dot{V}$O_2_ response to repeated bouts of heavy exercise, which shows that prior exercise results in faster “overall” $\dot{V}$O_2_ kinetics (as indicated by the reduced effective time constant). However, without partitioning the $\dot{V}$O_2_ response out into its distinct components discussed above, it could not be determined whether the speeding of the overall $\dot{V}$O_2_ kinetics following prior heavy exercise was due to a lower fundamental phase time constant ($\tau_{{\dot{V}{\text{O}}_{2} }}$), an increased fundamental phase amplitude or a reduced slow component. Figure 3 is a schematic of both of these putative responses to repeated bouts of heavy exercise. Later work confirmed the overall speeding of the $\dot{V}$O_2_ kinetics after prior heavy exercise, but demonstrated that this was not due to a reduction in $\tau_{{\dot{V}{\text{O}}_{2} }}$, but rather a reduction in the amplitude of the $\dot{V}$O_2_ slow component (Fig. 3B) [43, 44]. When $\dot{V}$O_2_ was given sufficient time to return to baseline following prior heavy exercise, then the amplitude of the fundamental phase $\dot{V}$O_2_ kinetics was increased during subsequent heavy exercise (Fig. 3B) [45]. These findings have since been replicated by multiple other experiments and research groups [2, 4, 7, 45–53]. Hence, these findings suggested that the characteristic effects of priming exercise on subsequent heavy exercise $\dot{V}$O_2_ kinetics were not to speed the $\dot{V}$O_2_ kinetics via a reduction in $\tau_{{\dot{V}{\text{O}}_{2} }}$, but rather, to increase and decrease the amplitudes of the fundamental and slow phases, respectively.

It was later demonstrated that there are several situations in which prior heavy exercise reduces $\tau_{{\dot{V}{\text{O}}_{2} }}$ in subsequent moderate or heavy exercise (Fig. 3A). For instance, prior heavy exercise performed in the supine or prone positions reduces $\tau_{{\dot{V}{\text{O}}_{2} }}$ during subsequent heavy exercise [2, 7, 16, 54, 55]. Moreover, prior heavy exercise reduced $\tau_{{\dot{V}{\text{O}}_{2} }}$ during subsequent moderate exercise in healthy elderly individuals [56], patients with type 2 diabetes mellitus [57, 58] and heart failure [59], and healthy individuals with initially slow kinetics [60], in contrast to healthy young active individuals. In each of these cases, $\dot{V}$O_2_ kinetics is markedly slowed in the initial bout of heavy warm-up exercise compared with $\dot{V}$O_2_ kinetics typically observed during upright cycle exercise in young healthy individuals. This suggests that when $\tau_{{\dot{V}{\text{O}}_{2} }}$ is greater in the control (i.e. unprimed) condition, there is a greater potential for subsequent speeding of fundamental phase $\dot{V}$O_2_ kinetics following prior heavy exercise. Indeed, this notion is supported by a wealth of more recent studies demonstrating that even in young healthy active individuals performing upright cycle exercise, $\tau_{{\dot{V}{\text{O}}_{2} }}$ may be reduced in subsequent moderate or heavy exercise by the performance of prior heavy exercise in a manner that is dependent upon the initial value of $\tau_{{\dot{V}{\text{O}}_{2} }}$ [7, 60–65]. Specifically, individuals with slower initial kinetics experience a greater reduction in $\tau_{{\dot{V}{\text{O}}_{2} }}$ following heavy-intensity priming exercise when compared with individuals with faster kinetics who experience little or no reduction in $\tau_{{\dot{V}{\text{O}}_{2} }}$ following priming.

**Fig. 4:**
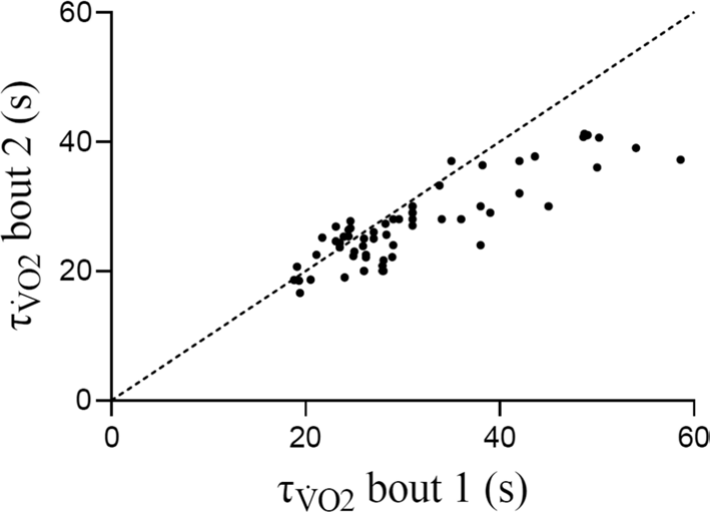
Time constant for the fundamental phase of pulmonary oxygen uptake kinetics ($\tau_{{\dot{V}{\text{O}}_{2} }}$) across 43 priming exercise studies published between 1996 and 2021 with the $\tau_{{\dot{V}{\text{O}}_{2} }}$ value in bout 2 plotted as a function of $\tau_{{\dot{V}{\text{O}}_{2} }}$ in bout 1. The values used were limited to studies addressing the effects of prior leg exercise on subsequent leg exercise; however, the intensities of the prior and criterion bouts of exercise varies between studies (heavy, severe and extreme) and multiple modes of exercise are included (upright and supine cycle exercise, supine and prone knee extensor exercise, and transitions from an elevated baseline work rate). For several studies, multiple groups are included, reflecting the different experimental conditions employed in these studies. In these cases, each experimental condition reflects a separate data point. Studies included are: [2–4, 6, 7, 16, 42–48, 50, 52–54, 56, 57, 59, 60, 62–65, 65, 66, 69, 83, 85, 89, 100, 101, 122, 169–171, 171, 179–185]. Note the tendency for data points to adhere closely to the line of identity up to $\tau_{{\dot{V}{\text{O}}_{2} }}$ values of approximately 25 s. After this point, the $\tau_{{\dot{V}{\text{O}}_{2} }}$ values in bout 2 become consistently lower than those observed in bout 1. The dashed line represents the line of identity

In Fig. 4, we conducted a retrospective analysis of 43 studies published between 1996 and 2021 on the effects of prior leg exercise on subsequent leg exercise. Studies were included only if the initial bout of exercise was above the lactate/gas exchange threshold (i.e. heavy, severe or extreme priming); however, the intensity of the subsequent bout of exercise could be moderate, heavy or severe. This analysis shows that when priming exercise is performed in studies with an average value for $\tau_{{\dot{V}{\text{O}}_{2} }}$ of 25 s or less in the non-primed condition, $\tau_{{\dot{V}{\text{O}}_{2} }}$ typically does not differ in the second bout of exercise. However, for groups of individuals with an average value for $\tau_{{\dot{V}{\text{O}}_{2} }}$ of 25 s or greater, $\tau_{{\dot{V}{\text{O}}_{2} }}$ is reduced in bout 2 compared with bout 1 in a manner that is proportional to the initial value for $\tau_{{\dot{V}{\text{O}}_{2} }}$.

In summary, the early studies examining the effects of prior heavy exercise on subsequent heavy-exercise $\dot{V}$O_2_ kinetics demonstrated an overall speeding of the $\dot{V}$O_2_ response. It was later shown that this was not due to a reduction in $\tau_{{\dot{V}{\text{O}}_{2} }}$, but rather to a reduction in the amplitude of the $\dot{V}$O_2_ slow component and an increase in the fundamental phase $\dot{V}$O_2_ amplitude. However, subsequent studies have demonstrated that $\tau_{{\dot{V}{\text{O}}_{2} }}$ during subsequent moderate or heavy exercise may also be reduced following priming [56, 60, 66], with the magnitude of this reduction being negatively related to the initial value of $\tau_{{\dot{V}{\text{O}}_{2} }}$ (Fig. 4). As the magnitude of this effect decreases with $\tau_{{\dot{V}{\text{O}}_{2} }}$ values < 25 s, it may become more difficult or impossible to detect priming-induced changes in $\tau_{{\dot{V}{\text{O}}_{2} }}$ from pulmonary gas exchange measurements, thus accounting for the discrepancies between earlier and more recent studies.

## Mechanisms Underpinning the Priming Effect

### Role of Lactic Acidosis in Mediating the Priming Effect

Heavy exercise results in the accumulation of lactate in muscle, which is known to cause vasodilation and a right shift of the oxyhaemoglobin dissociation curve [1, 67, 68]. Gerbino et al. [1] thus reasoned that this systemically elevated lactate level following priming would have resulted in lactate-mediated vasodilation and a Bohr effect within the periphery, which would have in turn increased convective and diffusive O_2_ supply to the working muscles and reduced regional perfusion heterogeneity [1]. However, various follow-up studies argued against a key determining role for lactic acidosis-mediated enhancements to muscle O_2_ delivery. First, the characteristic priming effect can occur with prior moderate exercise in some situations, without the accumulation of lactate. Faisal et al. [69] demonstrated that both prior moderate (i.e. where no lactate accumulation occurs) and heavy exercise were capable of reducing $\tau_{{\dot{V}{\text{O}}_{2} }}$ during subsequent heavy exercise, and that the magnitude of this effect was the same for both intensities. Additionally, the fall in muscle microvascular PO_2_ kinetics in the rat hindlimb was accelerated at the onset of a second bout of contractions in the absence of lactic acidosis [70]. Moreover, the magnitude of the priming effect on the $\dot{V}$O_2_ fundamental and slow-phase amplitudes is either substantially reduced or absent when the prior exercise is performed in a different muscle group than the initial bout [48, 51, 71]. As systemic lactate levels were similar following both prior arm and leg exercise, this latter finding argues against the hypothesis that the priming effect is mediated via changes in the peripheral O_2_ supply because of lactic acidosis. Finally, square-wave arterial infusions of lactate of 10 mM in the canine hindlimb model failed to produce any discernible effect on the $\dot{V}$O_2_ slow component [72], a finding that is difficult to reconcile with an important role for lactate in producing the effects of priming, at least on the $\dot{V}$O_2_ slow component. Hence, given that the priming effect can occur in situations with no systemic lactic acidosis and that an arterial lactate infusion does not appreciably alter $\dot{V}$O_2_ kinetics, it is unlikely that residual lactic acidosis persisting after prior exercise, and its influence on microvascular O_2_ supply, is causally implicated in this effect in any quantitatively large manner.

### Role of Increased Muscle Temperature

An increased muscle temperature could contribute to alterations in $\dot{V}$O_2_ kinetics following priming by speeding the rate-limiting reactions associated with intracellular oxidative phosphorylation, and/or indirectly via an increased muscle O_2_ delivery via a rightward shift of the oxyhaemoglobin dissociation curve. Moreover, it has been suggested that the $\dot{V}$O_2_ slow component might be related to elevated muscle temperature via the Q_10_ effect (i.e. the effect of temperature on the metabolic rate), which would imply a reduction in energetic efficiency at higher muscle temperatures [73, 74].

Indeed, muscle temperature can directly affect the speed of $\dot{V}$O_2_ kinetics at the onset of muscle contractions. In isolated single muscle fibres (*Xenopus laevis*), an increase in myofibre temperature from 20 to 25 °C resulted in a speeding of intracellular PO_2_ kinetics and a greater decline in intracellular PO_2_ (i.e. akin to the observation of a faster fundamental phase kinetics and increased amplitude in humans with priming in vivo) [75]. An earlier study by Shiojiri et al. [76] observed that reducing muscle temperature from 36.8 to 30.2 °C via cold water immersion slowed pulmonary $\dot{V}$O_2_ kinetics during subsequent moderate exercise in healthy inactive human subjects, consistent with a role for muscle temperature regulating muscle $\dot{V}$O_2_ at exercise onset. However, an increase in muscle temperature (i.e. from 36 to 39 °C) resulted in a slightly but significantly *reduced* $\dot{V}$O_2_ slow component [77], which is inconsistent with the hypothesised action of the Q_10_ effect.

However, most studies performed on humans have failed to produce any priming-like effects on $\dot{V}$O_2_ kinetics via passive heating. For instance, Koppo et al. [78] utilised passive heating to bring about the same degree of elevation in muscle temperature as that produced via prior exercise, and showed that passive heating had no impact upon any aspect of the pulmonary $\dot{V}$O_2_ kinetics [78]. Additionally, Ingjer and Strømme [79] and Burnley et al. [46] showed that raising muscle temperature by ~ 3 °C did not change pulmonary $\dot{V}$O_2_ kinetics compared to a normothermic control condition. Rates of muscle heat production did not differ between two repeated bouts of supramaximal exercise despite a small (0.5 °C) increase in muscle temperature in the second bout [80, 81]. Finally, utilising muscle biopsies during heavy-intensity exercise both with and without prior heating, Gray et al. [82] found that elevated muscle temperature caused greater muscle PCr degradation and anaerobic ATP turnover without any concomitant alterations in $\dot{V}$O_2_ kinetics [82], effects somewhat unlike those typically observed with prior exercise. In summary, whilst there is a clear role for muscle temperature in modulating the rate of adjustment of muscle mitochondrial respiration in vitro, studies conducted on humans performing exercise in vivo with elevated muscle temperature have failed to produce effects on pulmonary $\dot{V}$O_2_ kinetics that resemble those of priming. In general, it seems likely that any increase in muscle temperature brought about by prior exercise is too small to account for the priming effect.

### Role of Enhanced O_2_ Delivery in Mediating the Priming Effect

There is ample evidence that prior exercise enhances convective O_2_ delivery. For instance, heart rate, and hence bulk blood flow, is elevated following priming exercise both at baseline and during the subsequent exercise bout [2, 45, 53], and heart rate kinetics are speeded following prior heavy exercise [2]. Estimated cardiac output has also been shown to be elevated at baseline and during the early phase of the subsequent exercise bout following priming [52, 53], and cardiac output kinetics are also speeded [52]. Moreover, there is direct evidence of greater muscle blood flow at the onset of a second bout of contractions [80, 81, 83, 84]. Changes thereof following priming would enhance convective O_2_ delivery to working muscle during subsequent exercise.

Studies utilising near-infrared spectroscopy have shown that indices of muscle microvascular oxygenation are enhanced following prior exercise. For instance, both total[heme] and muscle O_2_ saturation are elevated following priming both at the onset and throughout subsequent exercise [2, 7, 45, 51, 63, 66, 83, 85]. The primary determinant of effective muscle diffusive capacity (*D*O_2_) is the number of red blood cells adjacent to the contracting myocytes at any given instant [86–88]. Hence, increased total[heme] and muscle O_2_ saturation suggest both an enhanced muscle *D*O_2_ and a higher blood-myocyte O_2_ diffusion driving pressure following priming, respectively. Priming exercise also reduces the spatial heterogeneity of muscle deoxygenation [89, 90], suggesting that priming reduces regional heterogeneity of perfusion between distinct muscle regions. Moreover, the increase in total[heme] following priming is strongly correlated with the priming-induced reduction in the $\dot{V}$O_2_ slow component amplitude [85]. Indeed, interventions that enhance O_2_ delivery, such as hyperoxia, typically reduce the amplitude of the $\dot{V}$O_2_ slow component [42, 91], or slow its rate of development [92, 93], whereas interventions that impair O_2_ delivery tend to increase its amplitude [54, 94, 95]. Hence, the increase in muscle microvascular haematocrit and O_2_ delivery following priming may be causally related to the priming-induced reduction in the $\dot{V}$O_2_ slow component amplitude.

There is also indirect evidence that enhanced O_2_ delivery may be related to priming-induced reductions in $\tau_{{\dot{V}{\text{O}}_{2} }}$. O_2_ delivery is generally considered to constrain $\dot{V}$O_2_ kinetics during supine exercise [54, 94, 95] and various clinical conditions (e.g. type 2 diabetes [57, 96]), and in some studies, prior exercise has been shown to reduce $\tau_{{\dot{V}{\text{O}}_{2} }}$ during supine, but not upright exercise [2, 7, 54] and in diabetic patients but not healthy controls [58] (although see [57] for alternative findings from the same group). In the canine hindlimb model, there was a concomitant speeding of muscle blood flow on-kinetics and a reduction in $\tau_{{\dot{V}{\text{O}}_{2} }}$ at the onset of a second bout of muscle contractions [97]. Moreover, some (although not all, c.f. [3, 6]) studies report slower muscle deoxygenation kinetics following priming [64, 89], a finding consistent with improved muscle O_2_ delivery.

Several studies have been conducted on the effects of prior exercise on the ratio between muscle deoxygenation (i.e. measured by near-infrared spectroscopy as deoxy[heme]) and pulmonary $\dot{V}$O_2_ kinetics. The Fick Equation dictates that muscle O_2_ extraction is dependent upon the ratio of muscle blood flow to muscle $\dot{V}$O_2_. As deoxy[heme] reflects muscle O_2_ extraction within the interrogated region, the ratio of deoxy[heme]/$\dot{V}$O_2_ has been interpreted to reflect changes in muscle microvascular blood flow [8, 9, 29, 30, 64–66, 98]. Utilising this method, Murias et al. [64] demonstrated that a prior bout of heavy-intensity exercise reduced $\tau_{{\dot{V}{\text{O}}_{2} }}$ during subsequent moderate exercise. This speeding of the fundamental $\dot{V}$O_2_ kinetics was observed alongside a concomitant reduction in the deoxy[heme]/$\dot{V}$O_2_ ratio in the subsequent moderate exercise, and the reduction in the deoxy[heme]/$\dot{V}$O_2_ ratio between bouts 1 and 2 was correlated with the reduction in $\tau_{{\dot{V}{\text{O}}_{2} }}$ between bouts 1 and 2 [64]. A later study by the same group showed that prior heavy exercise did not reduce $\tau_{{\dot{V}{\text{O}}_{2} }}$ or the deoxy[heme]/$\dot{V}$O_2_ ratio during subsequent moderate exercise in hypoxia [65]. This was interpreted to indicate that the priming-induced increases in muscle O_2_ delivery were effectively blunted by hypoxia, thereby preventing a reduction of $\tau_{{\dot{V}{\text{O}}_{2} }}$ in the second bout [65]. Collectively, these results have been taken as evidence to indicate that improvements in O_2_ availability at the onset of the second bout of exercise are implicated in changes in $\tau_{{\dot{V}{\text{O}}_{2} }}$ following priming exercise. However, interpretation of the deoxy[heme]/$\dot{V}$O_2_ ratio is complicated by the local muscle deoxygenation heterogeneity, total heme concentration and concomitant temporal changes in blood flow and metabolism, and hypoxia has been shown to alter intramuscular deoxygenation heterogeneity during the on-transient of exercise [99].

Despite the evidence reviewed above, there is evidence that improvements in O_2_ delivery are not necessary for the priming effect to occur. For instance, it has been shown that in cases where changes in leg blood flow kinetics do not occur following prior heavy exercise, the typical overall speeding of $\dot{V}$O_2_ kinetics in subsequent exercise persists [100, 101]. Although this does not rule out a potential role for improved regional distribution of O_2_, this suggests that alterations in convective O_2_ delivery at the level of the conduit artery are not responsible for the priming effect. Although a relatively blunt measure of muscle perfusion heterogeneity, the reduction in the spatial heterogeneity of muscle deoxygenation following priming also does not correlate with changes in either $\tau_{{\dot{V}{\text{O}}_{2} }}$ or the $\dot{V}$O_2_ slow component amplitude [7, 85, 89, 90], suggesting that improved spatial O_2_ distribution is not a key determinant of the priming effect. Similarly, microvascular PO_2_ kinetics were speeded at the onset of the second bout of contractions in rat spinotrapezius muscle [70] and single isolated muscle fibres [102, 103] without changes in baseline PO_2_. Although extrapolating results from the level of single fibre or muscle to the intact human performing dynamic exercise in vivo presents multiple challenges, collectively, these data suggest that it is at least possible for a speeding of $\dot{V}$O_2_ kinetics to occur subsequent to priming exercise without changes in the O_2_ delivery-to-utilisation ratio.

Muscle O_2_ extraction is greater for a given absolute or relative $\dot{V}$O_2_ in the supine versus upright positions [95]. If the priming-induced reduction in $\tau_{{\dot{V}{\text{O}}_{2} }}$ following priming during supine exercise [2, 54] was solely due to increased O_2_ availability, then fractional O_2_ extraction (measured by time-resolved near-infrared spectroscopy) should return to the values typically observed during upright exercise [7]. As expected, the amplitude of muscle deoxy[heme] kinetics and $\tau_{{\dot{V}{\text{O}}_{2} }}$ were greater in bout 1 in the supine compared with the upright position [7]. In bout 2, however, $\tau_{{\dot{V}{\text{O}}_{2} }}$ in the supine position was reduced to values similar as in the upright position, whereas muscle O_2_ extraction was unchanged, even after normalisation for muscle activity [7]. Although there was clear evidence that microvascular O_2_ availability was enhanced following priming in this study [7], the fact that fractional O_2_ extraction remained markedly elevated above that in the upright position after priming indicates that priming did not fully redress the impairment in O_2_ delivery induced by supine exercise. As $\tau_{{\dot{V}{\text{O}}_{2} }}$ did not differ between upright and supine exercise after priming, enhanced intracellular O_2_ utilisation (along with improvements in microvascular O_2_ availability) after priming must have contributed to the observed speeding of $\dot{V}$O_2_ kinetics. This study thus indicates that improvements in O_2_ delivery *alone* are not sufficient to explain the impact of priming on $\tau_{{\dot{V}{\text{O}}_{2} }}$, and that even in situations where O_2_ delivery is constrained and thus presumed to limit the speed of the $\dot{V}$O_2_ kinetics [104], an interaction between enhancements in both O_2_ delivery and utilisation most likely explains the priming effect on $\tau_{{\dot{V}{\text{O}}_{2} }}$.

Studies addressing the control of $\dot{V}$O_2_ kinetics under standard (i.e. non-primed) conditions are insightful for understanding the priming effect because if any given factor (i.e. O_2_ delivery) is not rate limiting under standard conditions, then its upregulation via priming would be unlikely to result in a subsequent speeding of the $\dot{V}$O_2_ response. In the highly oxidative canine hindlimb model, pump perfusion of the skeletal muscle to rates of O_2_ delivery well above the steady-state requirements during muscle contractions [105] and enhancing peripheral O_2_ diffusion via pharmacologically right shifting the oxyhaemoglobin curve [106] do not affect $\tau_{{\dot{V}{\text{O}}_{2} }}$. Conversely, graded reductions in O_2_ delivery in this model produce linear increases in $\tau_{{\dot{V}{\text{O}}_{2} }}$ [107]. In humans performing upright cycle exercise at sea level, interventions that enhance O_2_ delivery, such as hyperoxia [42, 91, 92, 108, 109], erythropoietin administration [110] and lower body positive pressure [111], do not affect $\tau_{{\dot{V}{\text{O}}_{2} }}$ (c.f. [112]). These findings therefore indicate that muscle O_2_ delivery does not constrain $\tau_{{\dot{V}{\text{O}}_{2} }}$ under standard conditions. It is important to note, however, that one study did find that erythropoietin administration speeded $\dot{V}$O_2_ kinetics in a group of trained cyclists [112]. However, the presence of a slow component in some subjects, and the observation that the $\tau_{{\dot{V}{\text{O}}_{2} }}$ values measured in this study were unusually large for subjects with such large $\dot{V}$O_2max_ values (i.e. $\tau_{{\dot{V}{\text{O}}_{2} }}$ of 36 s for $\dot{V}$O_2max_ of 64–65 mL.kg^−1^.min^−1^, c.f. [113]), suggest that inclusion of a slow component in the modelled fits may have influenced this finding. Hence, whilst it is clear that in the majority of studies the performance of prior exercise enhances O_2_ delivery, and there is one instance in the literature where removal of the increased O_2_ delivery engendered by priming abolished the priming effect [65], the weight of evidence indicates that improvements in O_2_ delivery are coincident with, but not necessarily responsible for, changes in $\tau_{{\dot{V}{\text{O}}_{2} }}$ subsequent to priming. There is some evidence that enhancements to O_2_ delivery following priming may be related (i.e. either directly or indirectly via effects on motor unit recruitment and cellular energy status) to the effects of priming on the $\dot{V}$O_2_ slow component, but the evidence that $\tau_{{\dot{V}{\text{O}}_{2} }}$ after priming exercise can be affected by improved O_2_ delivery in healthy young subjects is much weaker. In support of this, a recent in silico modelling study demonstrated that $\tau_{{\dot{V}{\text{O}}_{2} }}$ was weakly dependent on intracellular O_2_ levels with $\tau_{{\dot{V}{\text{O}}_{2} }}$ values between 20 and 30 s [114]. This suggests that in young healthy subjects, improvements in O_2_ delivery consequent to priming likely have a small quantitative contribution to changes in $\tau_{{\dot{V}{\text{O}}_{2} }}$. However, in populations with larger $\tau_{{\dot{V}{\text{O}}_{2} }}$ values, such as the healthy elderly and populations with chronic diseases, improvements in O_2_ delivery consequent to priming, along with changes in intracellular O_2_ utilisation, may play a larger role in the control of $\tau_{{\dot{V}{\text{O}}_{2} }}$ [104]. Hence, a role for improved O_2_ delivery subsequent to priming in reducing $\tau_{{\dot{V}{\text{O}}_{2} }}$ in such populations is likely. Further human studies on priming exercise that adequately control for O_2_ utilisation and motor unit recruitment are necessary to further clarify this issue.

### Role for Altered Motor Unit Recruitment

One mechanism that has been strongly implicated in the priming effect is altered motor unit recruitment patterns. The evidence underpinning this mechanism is drawn largely from measurements using bipolar surface electromyography (EMG), which has important limitations in interpreting the output of the motor unit pool [115]. Nevertheless, the conceptual basis of the role of motor unit recruitment patterns is straightforward (see [116] for a more detailed review). Briefly, the fundamental $\dot{V}$O_2_ amplitude represents the initial target amplitude, or the anticipated steady-state response that can only be achieved during moderate-intensity exercise. The subsequent emergence of the $\dot{V}$O_2_ slow component represents an elevated O_2_ requirement in response to neuromuscular fatigue [117–119] (although for an alternative interpretation, see [120, 121]). One factor that drives $\dot{V}$O_2_ above the anticipated steady state during heavy- and severe-intensity exercise is thought to be the recruitment of additional motor units and the resulting metabolism of the fibres associated with them [34]. In this scheme, priming exercise has the effect of increasing motor unit recruitment at exercise onset, resulting in the recruitment of fewer motor units as exercise progresses. The result of this would be an increased fundamental $\dot{V}$O_2_ amplitude and a reduced $\dot{V}$O_2_ slow component amplitude. Moreover, the fact that more motor units are activated at the onset of exercise would mean that the rate of fatigue within each recruited fibre would be reduced, potentially enhancing exercise tolerance [5, 6, 122, 123].

Although the above model is plausible, the evidence in direct support of it is limited to the analysis of the surface EMG. A number of studies have sampled the EMG from the knee and/or hip extensors (i.e. those muscles contributing to power production), with mixed results. The first to do so found no evidence of increased integrated EMG (iEMG) coincident with the $\dot{V}$O_2_ slow component, and no statistically significant difference in iEMG between the first and second bouts of heavy exercise [50]. However, the sampling of a single muscle and the normalisation of the EMG signal to the value at the end of exercise may have limited the ability to identify a detectable change following prior heavy exercise.

Burnley et al. [45] demonstrated that prior heavy exercise increased the fundamental $\dot{V}$O_2_ amplitude and reduced the $\dot{V}$O_2_ slow component. Crucially, the iEMG sampled from three muscles (vastus lateralis, vastus medialis and the gluteus maximus) showed a ~ 19% increase in the first 2 min of the second bout of heavy exercise, coincident with the increase in the fundamental amplitude. These authors reasoned that prior contractile activity altered the recruitment threshold of higher order motor units during subsequent exercise, allowing more motor units to be recruited from exercise onset and lessening the requirement for the recruitment of additional motor units as exercise progressed. This is an attractive hypothesis because it could account for both the increased fundamental $\dot{V}$O_2_ amplitude and reduced slow component amplitude observed following priming.

Support for the above hypothesis was generated by Layec et al. [124], who demonstrated using ^31^P-MRS and EMG during repeated bouts of contractions that prior exercise increased motor unit recruitment during subsequent exercise. In turn, this was associated with an increased oxidative ATP cost and decreased ATP production from PCr breakdown and glycolysis during the early phase of the exercise bout, and a reduced global O_2_ cost during the latter stages of exercise [124]. Hence, these findings lend direct experimental support for the notion that prior exercise alters motor unit recruitment patterns during subsequent exercise, which in turn produces positive effects on muscle energetics that would postpone fatigue and the recruitment of additional motor units. The findings of other studies utilising EMG analysis have, however, been mixed: Bailey et al. [6] demonstrated an increase in iEMG during repeated bouts of severe-intensity exercise, whereas Tordi et al. [53] showed no change following repeated bouts of sprint exercise.

Whilst the notion that the priming-induced increase in the fundamental $\dot{V}$O_2_ amplitude is caused by increased motor unit recruitment is logical, and has some support in the literature, it does present further difficulties in terms of the interpretation of motor unit activity: if the same power output is achieved by the recruitment of additional motor units, how does previous activity reduce the recruitment threshold of higher order motor units, which may not have been activated in the first bout? How does the central nervous system control descending drive to ensure the correct motor units are recruited and are firing at the right frequency? Bipolar EMG does not provide an accurate measure of neural drive to the muscle or motor unit recruitment during exercise as the small volume of muscle sampled and the interference pattern of the motor unit action potential train obscure the true motor unit output, largely owing to amplitude summation and/or cancellation [125]. The influence of motor unit recruitment, if any, in the effects of prior heavy exercise must await the application of more advanced techniques, such as high-density surface EMG and signal decomposition [126].

Correlational studies are, in general, supportive of a link between fibre-type composition and the amplitude of the $\dot{V}$O_2_ slow component [39, 127–131]. Alterations in pedalling cadence, which are predicted to increase recruitment of higher order motor units, tend to increase the $\dot{V}$O_2_ slow component amplitude [132, 133]. Selective glycogen depletion of either type I or II fibres alters the amplitudes of the fundamental phase $\dot{V}$O_2_ kinetics [134, 135] or the $\dot{V}$O_2_ slow component [134, 136] (cf. [137]), respectively. Furthermore, the recruitment of additional higher order motor units during heavy exercise occurs in close temporal association with the development of the $\dot{V}$O_2_ slow component [37, 138]. Finally, the increase of the iEMG signal throughout heavy exercise has been demonstrated to correlate closely with the $\dot{V}$O_2_ slow component amplitude [139, 140]. In summary, single muscle fibre studies evincing changes in $\tau_{{\dot{V}{\text{O}}_{2} }}$ following priming likely rule out a role for motor unit recruitment in determining changes in this parameter. However, some evidence supports the notion that alterations in motor unit recruitment can partially account for changes in the fundamental and slow phases of the $\dot{V}$O_2_ kinetics subsequent to priming.

### Role for Enhanced Intracellular O_2_ Utilisation

There is substantial evidence supporting a role for enhanced intracellular O_2_ utilisation in underpinning certain aspects of the priming effect. Among the first to address a role for enhanced intracellular O_2_ utilisation was Hogan [103], who performed two repeated 3-min bouts of electrically stimulated contractions separated by 5 min of rest in *X. laevis* single myofibres. This model eliminates O_2_ availability or motor unit recruitment as confounding factors because *X. laevis* fibres lack myoglobin, *P*O_2_ surrounding the cell is uniform and isolated single fibres contract via electrical stimulation. In the second bout of contractions, the time delay before the decline in intracellular *P*O_2_ was reduced, leading to faster overall intracellular *P*O_2_ kinetics [103]. Consistently, Behnke et al. [70] demonstrated that microvascular *P*O_2_ kinetics were faster at the onset of a second bout of contractions in the rat hindlimb despite no changes in baseline *P*O_2_, suggesting that O_2_ delivery was not enhanced at the onset of a second bout of contractions. Both these studies implicate more rapid adjustments in intracellular O_2_ utilisation as a central determinant of the priming effect on $\tau_{{\dot{V}{\text{O}}_{2} }}$. Although extrapolating results from the level of a single fibre or muscle to the intact human performing dynamic exercise in vivo presents multiple challenges, these data suggest that it is at least possible for a speeding of $\dot{V}$O_2_ kinetics to occur subsequent to priming exercise without changes in the O_2_ delivery-to-utilisation ratio.

#### Role for Increased Mitochondrial Complex Activity and/or Availability of Substrate

An increase in pyruvate dehydrogenase (PDH) activity following priming has been suggested to alleviate the functional inertia regulating substrate flux into the Krebs’ cycle [61–63, 141–143]. This is because delayed PDH activation at exercise onset prevents sufficient acetyl group flux into the tricarboxylic acid (TCA) cycle. Priming could, therefore, overcome this limitation by increasing metabolic substrate availability [144]. Wüst and Stienen [145] observed that the priming effect was abolished in rat cardiac trabeculae when saturating concentrations of pyruvate were added to the superfusate, suggesting that PDH activity is likely involved in the mitochondrial priming effect. Moreover, Howlett and Hogan [143] found that dichloroacetate (DCA) administration, which activates the PDH complex, sped intracellular *P*O_2_ kinetics at the onset of contractions of the *X. laevis* single fibres. DCA administration also reduces PCr degradation and improved fatigue resistance in the canine hindlimb [146, 147] and human muscle [148]. Priming exercise was shown to lead to an accumulation of acetyl groups at the onset of subsequent exercise, reduced substrate-level phosphorylation and speeded overall $\dot{V}$O_2_ kinetics [144], Similarly, prior heavy exercise increased PDH activity and speeded $\dot{V}$O_2_ kinetics during subsequent moderate exercise in young [63][63] and old individuals, although changes in PDH activity did not correlate with changes in $\tau_{{\dot{V}{\text{O}}_{2} }}$ between bouts 1 and 2 [61, 62]. Hence, a cause-and-effect relationship remains elusive.

Utilising the canine hindlimb model, Grassi et al. [141] demonstrated that DCA administration accumulated acetyl groups that reduced muscle fatigue during subsequent exercise, but $\dot{V}$O_2_ kinetics were unaltered. As force declines with time with fatiguing contractions in this model, when $\dot{V}$O_2_ is normalised by force production, a $\dot{V}$O_2_ slow component becomes evident [149]. As fatigue was reduced with DCA in the study by Grassi et al. [141], this suggests that the DCA-induced activation of the PDH complex reduced the progression of the $\dot{V}$O_2_ slow component. Hence, this study is consistent with a role for an improvement in mitochondrial substrate availability consequent to improved PDH activation in producing the priming effect on the $\dot{V}$ O_2_ slow component.

However, DCA administration has failed to demonstrate any effect on $\dot{V}$O_2_ kinetics in humans. For instance, no effect of DCA on the fundamental phase $\dot{V}$O_2_ kinetics during moderate- [150], heavy- [151] and severe-intensity exercise [152] has been demonstrated in humans, despite a four- to eight-fold increase in PDH activation occurring in some studies [153]. DCA administration did not reduce either $\tau_{{\dot{V}{\text{O}}_{2} }}$ or the time constant for muscle PCr kinetics during heavy knee-extension exercise in humans, but rather reduced the amplitude of the fundamental $\dot{V}$O_2_ and PCr responses. Hence, although there is some evidence that PDH activation can elicit priming-like responses in some experimental preparations, all studies using a rigorous $\dot{V}$O_2_ kinetics analysis in humans have failed to find any impact of direct PDH activation via DCA on pulmonary $\dot{V}$O_2_ kinetics. However, studying the effects of an isolated aspect of mitochondrial metabolism in such a way is complex because the activities of all enzymes involved in oxidative metabolism influence the overall rate of mitochondrial ATP production to an extent that is dependent upon their individual flux control ratios [154]. Hence, if PDH is activated without a simultaneous increase in the activities of the enzymes of the electron transport system, for instance, it is unlikely that any effect on $\dot{V}$O_2_ kinetics would be observed. It therefore remains possible that enhanced substrate provision to the mitochondrial electron transport system consequent to greater PDH activation at exercise onset can account for some of the effects on priming exercise. However, without a method to simultaneously enhance PDH activity whilst ensuring that downstream activities of the TCA cycle or electron transport system are not limiting, it remains complex to study its isolated effects in vivo.

#### Role for Parallel Activation of ATP-Consuming and ATP-Producing Processes

Ultimately, the intracellular factors that mediate the priming effect are unknown. However, some suggestions may be made by considering the intracellular factors known to control muscle $\dot{V}$O_2_ at exercise onset, albeit speculative suggestions. Respiration in isolated mitochondria typically conforms to classical Michaelis–Menten kinetics in response to increasing ADP [155]. However, the relation between ADP and $\dot{V}$O_2_ in vivo is too steep to be described by a first-order model [156, 157], suggestive of a higher order allosteric activation model to explain the control of oxidative phosphorylation on transition to exercise [156] [157]. Indeed, the mitochondrial $\dot{V}$O_2_ response at contractile onset in single *X. laevis* myofibres is not a single exponential, but displays an initial slower “activation” phase, followed by a more rapid exponential increase towards steady state [158]. Importantly, single-fibre $\dot{V}$O_2_ kinetics revert to first-order in the recovery from contractions, suggesting that the activation processes that occur at exercise onset recover much more slowly at exercise offset [158]. It has been suggested that this distinct biphasic response is due to the parallel activation of ATP-consuming and ATP-producing processes at the onset of exercise, and that a cytosolic factor or regulatory mechanism such as calcium is likely responsible for this [159, 160].

**Fig. 5:**
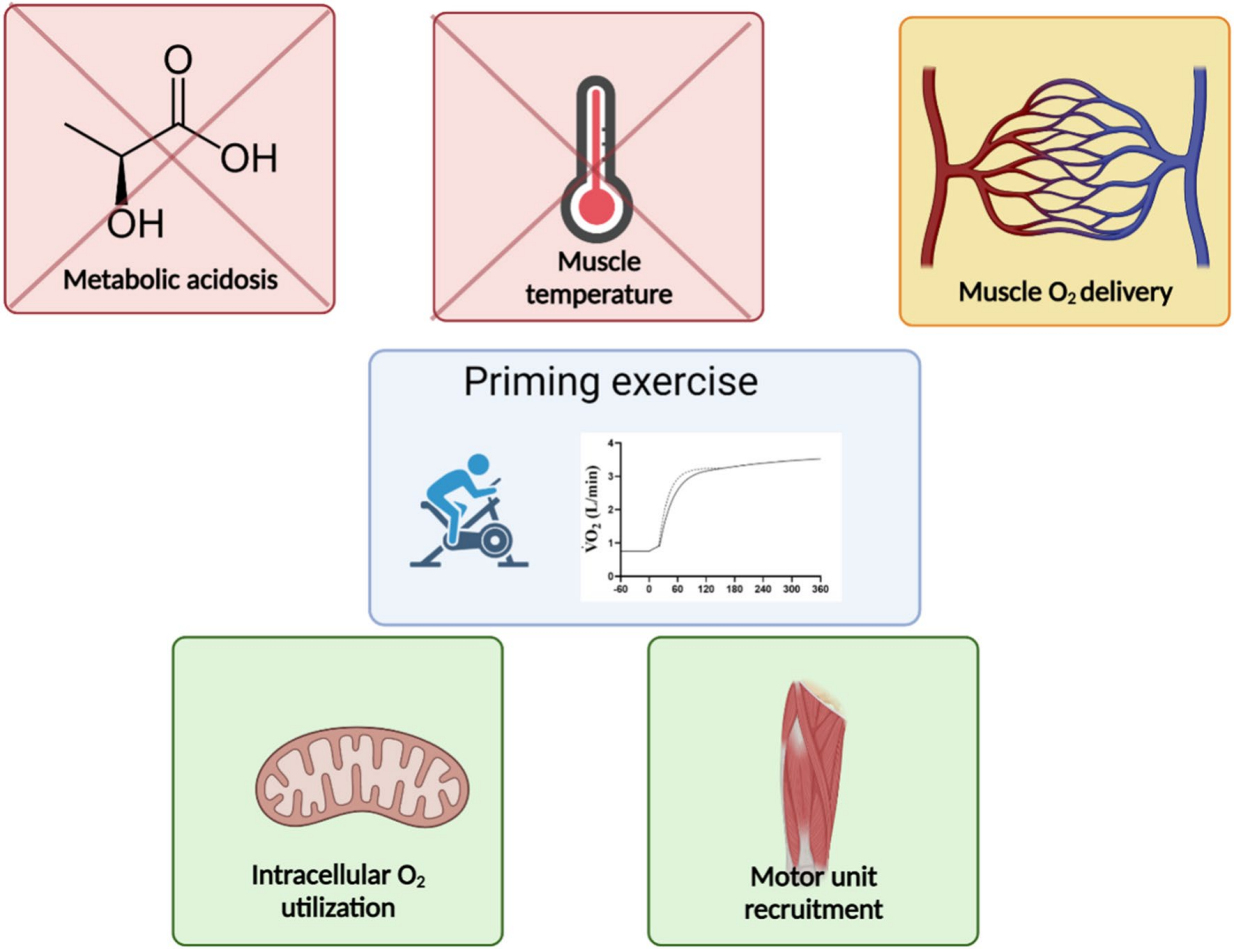
Schematic displaying the characteristic effects of prior heavy exercise on the pulmonary oxygen uptake ($\dot{V}$O_2_) kinetics and their mechanistic determinants. Green boxes indicate mechanisms with direct experimental support and no clear contradictory evidence. The amber box indicates mechanisms with some experimental evidence both for and against. Red crosses indicate mechanisms that have large amounts of evidence against them. See text for further details

One factor that could *potentially* mediate such parallel-activation processes is an accumulation of mitochondrial calcium. Indeed, mitochondrial calcium accumulation occurs at the start of contractions in skeletal [161] and cardiac [162] muscle, which are more rapid compared with relatively slow extrusion kinetics at the end of isolated stimulated contractions. Mitochondrial calcium levels remain elevated in frog myocytes for up to 1 h after contractions [163], and calcium accumulation in the mitochondrial matrix modulates the activity of multiple mitochondrial enzymes and complexes involved in ATP production [164]. Hence, a calcium-mediated increase in mitochondrial enzyme activity at the onset of a second bout of exercise, due to persistent elevations in calcium following the first bout of contractions [163], could serve to accelerate mitochondrial $\dot{V}$O_2_ kinetics. Such a mechanism could potentially be capable of mediating the effect of priming on $\tau_{{\dot{V}{\text{O}}_{2} }}$ often observed in humans performing exercise in vivo. Hence, studying the role of calcium in determining the priming effect (in single fibres, in humans using serial muscle biopsies or in computer simulation studies) might prove a fruitful area for future research. Although speculative, the hyperbolic relationship between each-step activation of oxidative phosphorylation and $\tau_{{\dot{V}{\text{O}}_{2} }}$ [165] might also explain why it is less likely that $\tau_{{\dot{V}{\text{O}}_{2} }}$ will be reduced by priming in individuals with low initial $\tau_{{\dot{V}{\text{O}}_{2} }}$ values. In such individuals, much larger increases in each-step activation would be required to elicit further reductions in $\tau_{{\dot{V}{\text{O}}_{2} }}$, meaning that any mitochondrial calcium accumulation following priming may be insufficient to speed $\dot{V}$O_2_ kinetics in such individuals. Alternatively, the intrinsic measurement error in whole body/muscle $\dot{V}$O_2_ (together with the buffering capacity of myoglobin) might contribute as well. That priming was also observed in NAD(P)H kinetics in frog muscles [102] and isolated trabeculae [145] supports this notion. Hence, it seems that intracellular mechanisms may be central to the observed changes to $\tau_{{\dot{V}{\text{O}}_{2} }}$ following priming; however, a great deal of further investigation is clearly required to understand how these mechanisms operate in vivo. A schematic displaying the putative mechanistic determinants of the priming effect is displayed in Fig. 5.

## Impact of Priming on Endurance Performance

Whether exercise performance is enhanced via priming exercise appears to depend critically upon the relative intensity of the prior and subsequent exercise relative to critical power and the intervening recovery duration. For instance, whilst there are many documented instances where priming has improved subsequent exercise performance [2, 4–7, 123, 166], there are also instances where it either does not affect [122, 167, 168] or even reduces [6, 169, 170] subsequent exercise tolerance. When interpreting the data that has failed to show any improvement in exercise tolerance following prior exercise, it is important to bear in mind that there are substantial between-study differences in the intensities of the prior exercise and criterion exercise bouts and in the intervening recovery durations.

The optimal intensity and recovery duration of exercise performed prior to competition remain unclear. Although some data indicate that prior sprint- or severe-intensity exercise impairs subsequent performance [169, 170], prior severe-intensity exercise has been shown to accelerate subsequent $\dot{V}$O_2_ kinetics to a greater extent than heavy exercise [6], and these effects persist for up to 45 min [171]. Bailey et al. [6] observed that prior heavy exercise (Δ40%, i.e. 40% of the difference between the gas exchange threshold and $\dot{V}$O_2_ max) did not influence subsequent severe-intensity (i.e. Δ80%) exercise tolerance when the intervening recovery duration was 3, 9 or 20 min long [6]. However, prior severe-intensity exercise (i.e. Δ70%) impaired subsequent severe-intensity exercise tolerance when recovery was 3 min, but improved subsequent exercise performance when recovery was 9 (15% improvement) and 20 min (30% improvement) [6].

A critical confounding factor in the interpretation of this study is that the Δ% between the gas exchange threshold and $\dot{V}$O_2_ max does not account for inter-individual differences at which critical power occurs. Critical power represents the power asymptote of the hyperbolic relationship between external power and the tolerable duration of exercise, lies between the gas exchange threshold and $\dot{V}$O_2_ max (i.e. approximately Δ50%, but with considerable inter-individual variation), and delineates heavy- from severe-intensity exercise [20, 172–174]. During exercise performed above critical power, exercise tolerance is a function of W’, the curvature constant of the power-duration curve that represents a fixed quantity of work performable above critical power [20, 172–174]. Hence, in studies that have not also determined the parameters of the power-duration relationship (or any other means to determine the boundary between heavy- and severe-intensity domains), it becomes complex to interpret the impact of prior exercise on exercise performance because the prior and subsequent exercises are performed at different intensities relative to critical power and differ in their rates of W′ depletion, which likely explains the seemingly divergent results between studies.

Indeed, studies that have assessed the impact of severe-intensity priming on exercise performance whilst also determining the power–duration relationship have shown contradictory findings with Bailey et al. [6]. Ferguson et al. [169] observed a substantial reduction in severe-intensity exercise tolerance 15 min subsequent to a prior bout of severe-intensity exercise. Burnley et al. (4) also found that prior severe-intensity exercise had no effect on subsequent exercise tolerance, whereas prior heavy exercise enhanced it. Whilst there may be an ergogenic benefit of prior severe-intensity exercise if recovery durations are extended beyond 10 min [6], this is not a consistent finding, especially when the power–duration relationship is accounted for [4, 169]. Prior heavy-intensity exercise, producing modest elevations in blood lactate concentration (i.e. 2–3 mmol.L^−1^), appears to result in more consistent improvements in subsequent exercise performance [2–4, 122].

**Fig. 6:**
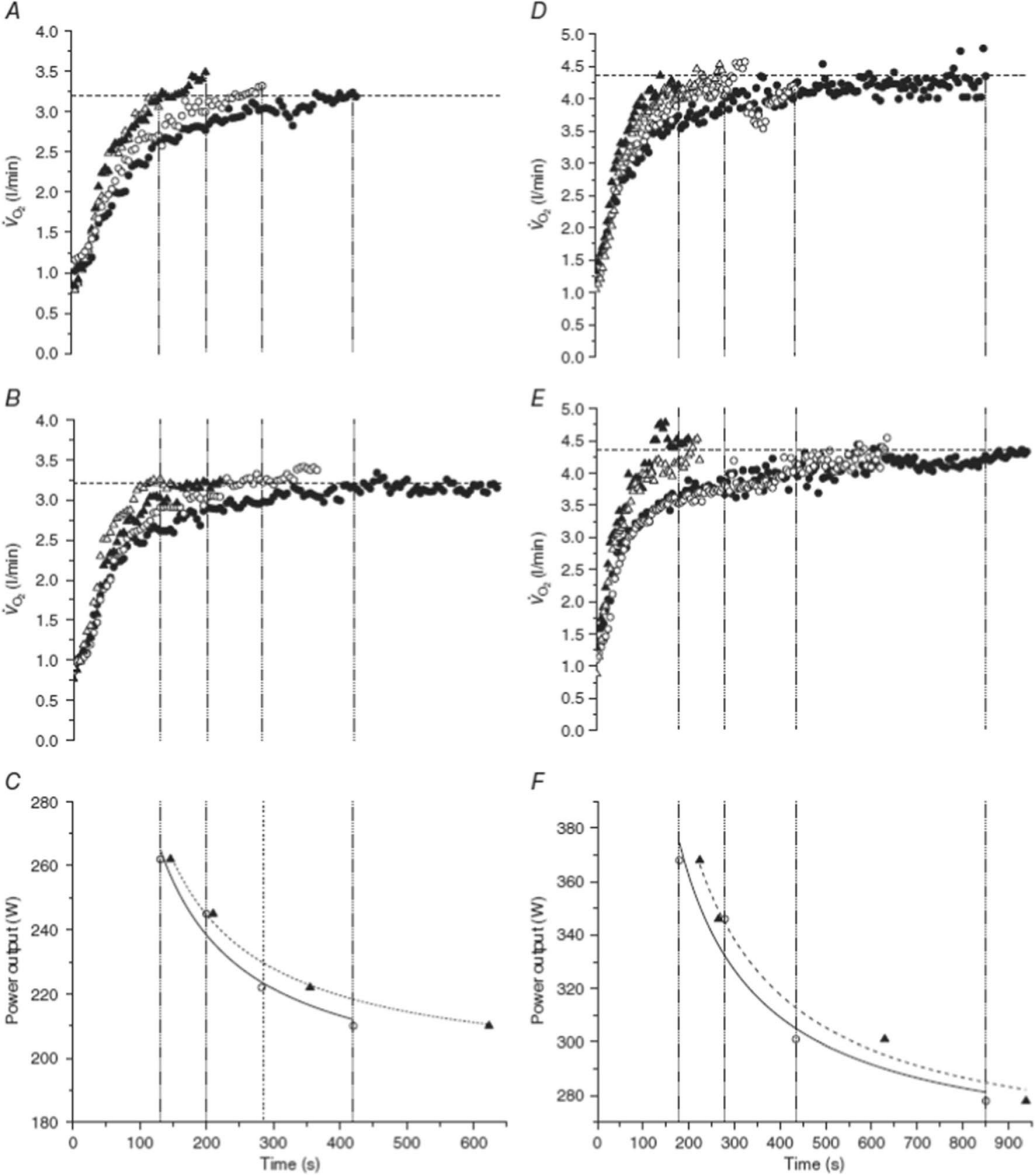
Two representative subjects showing the effects of priming exercise on pulmonary oxygen uptake ($\dot{V}$O_2_) responses and the power–duration relationship in situations where priming reduces the fundamental phase time constant ($\tau_{{\dot{V}{\text{O}}_{2} }}$) is reduced (**A–C**) versus situations where the fundamental phase amplitude is increased and the slow component is reduced (**D–F**). In **A** and **D**, pulmonary $\dot{V}$O_2_ responses are displayed in control conditions without prior exercise for two different subjects. For the subject depicted in (**A**), priming induces a reduction in $\tau_{{\dot{V}{\text{O}}_{2} }}$ (**B**), and consequently, an increase in critical power (**C**). For the subject depicted in (**D**), priming induces an increase in the fundamental $\dot{V}$O_2_ amplitude and a reduction in the amplitude of the slow component (**E**), the result of which is to increase W´ (**F**). Exhaustive exercise bouts were conducted at four severe-intensity work rates, with WR1 reflecting the lowest power output and WR4 reflecting the highest power output. In panels **A**, **B**, **D** and **E**, filled circles reflect WR 1; open circles reflect WR 2; filled triangles reflect WR 3; and open triangles reflect WR 4. Dashed horizontal lines represent each subject’s maximal $\dot{V}$O_2_, whereas the dotted vertical lines represent the time to task failure in the control (unprimed) condition. In (**C**, **F**), open and closed symbols represent the unprimed and primed conditions, respectively. Reproduced from Goulding et al. [2], with permission

How exercise performance is enhanced following prior exercise also appears to depend upon the manner in which the $\dot{V}$O_2_ kinetics are altered. For example, in studies in which there is no alteration in $\tau_{{\dot{V}{\text{O}}_{2} }}$ but an increase in the fundamental $\dot{V}$O_2_ amplitude and a decrease in the $\dot{V}$O_2_ slow component amplitude, W′ is typically increased, with no change in critical power [4, 5, 169]. Conversely, when $\tau_{{\dot{V}{\text{O}}_{2} }}$ is reduced by prior exercise with no change in the fundamental or slow component $\dot{V}$O_2_ amplitudes, critical power tends to increase, with no change [2] or a decrease [3] in W′. Hence, whether critical power or W′ are improved following prior exercise appears to depend critically upon whether $\tau_{{\dot{V}{\text{O}}_{2} }}$ or the amplitudes of the fundamental and/or slow phases of the $\dot{V}$O_2_ response are affected (Fig. 6). This observation has significant implications for the ergogenic potential of priming to enhance performance in any given endurance event. Individuals with rapid $\dot{V}$O_2_ kinetics (i.e. $\tau_{{\dot{V}{\text{O}}_{2} }}$ < 25 s) are unlikely to display any reduction in $\tau_{{\dot{V}{\text{O}}_{2} }}$ following priming [7, 60, 64], and hence the ergogenic effects will be restricted to improvements in W′. As W′ is only utilised during supra-critical power exercise, we speculate that individuals with high aerobic fitness will only benefit from priming exercise in events with a duration between 2 and 30 min, i.e. exercise above critical power, and hence an increase in W′ following priming would serve to increase the amount of work that an athlete can complete in such events. However, people with larger initial values for $\tau_{{\dot{V}{\text{O}}_{2} }}$ are more likely to reduce $\tau_{{\dot{V}{\text{O}}_{2} }}$ following priming [7, 60, 64], which will increase critical power [2, 3, 93, 175, 176]. $\tau_{{\dot{V}{\text{O}}_{2} }}$ is hyperbolically related to $\dot{V}$O_2_ max [113, 158, 177], and hence it is likely that untrained individuals with a low $\dot{V}$O_2_ max would experience a reduction in $\tau_{{\dot{V}{\text{O}}_{2} }}$ following priming. Hence, for untrained or recreationally active individuals, we anticipate that priming enhances performance during events of > 30 min in duration owing to an increase in critical power [2, 3, 93, 175, 176], where critical power sets the upper limit for sustainable exercise performance [172, 178]. Future research is required to test these hypotheses.

## Conclusions

Overall, $\dot{V}$ O_2_ kinetics are affected following prior heavy exercise due to a lower $\tau_{{\dot{V}{\text{O}}_{2} }}$, an increase in the fundamental phase $\dot{V}$ O_2_ amplitude and/or a decrease in the slow component amplitude, depending upon the training status and mode of exercise. An elevated muscle temperature, residual metabolic acidosis and improvements in O_2_ availability are likely not sufficient to completely explain the priming effect. Rather, alterations in motor unit recruitment patterns and an upregulation of intracellular O_2_ utilisation most likely explain the characteristic effects of priming on $\dot{V}$O_2_ kinetics. The manner in which exercise performance is influenced by prior exercise depends on the subject training status, as this affects the manner in which $\dot{V}$O_2_ kinetics are altered by prior exercise. We hypothesise that aerobically trained individuals are more likely to evince an enhanced W′ following prior exercise, and as such, events spanning 2–30 min in duration stand to benefit from this intervention. In untrained or recreationally active individuals, we propose that critical power is more likely to increase following priming, with no change in W′. Hence, events lasting > 30 min in duration, where critical power sets the upper limit for sustainable performance, are more likely to benefit from the performance of prior exercise.
